# Strengthening resilience to reduce HIV risk in Indian MSM: a multicity, randomised, clinical efficacy trial

**DOI:** 10.1016/S2214-109X(20)30547-7

**Published:** 2021-04

**Authors:** Steven A Safren, Beena Thomas, Katie B Biello, Kenneth H Mayer, Shruta Rawat, Alpana Dange, C Andres Bedoya, Sunil Menon, Vivek Anand, Vinoth Balu, Conall O’Cleirigh, Lynne Klasko-Foster, Dicky Baruah, Soumya Swaminathan, Matthew J Mimiaga

**Affiliations:** Department of Psychology, University of Miami, Coral Gables, FL, USA (Prof S A Safren PhD); Fenway Institute, Fenway Health, Boston, MA, USA (Prof S A Safren, Prof K H Mayer MD, C O’Cleirigh PhD, Prof M J Mimiaga ScD); National Institute for Research in Tuberculosis, Indian Council of Medical Research, Chennai, India (B Thomas PhD, V Balu, S Swaminathan MD); Department of Behavioural and Social Health Sciences and Epidemiology, Brown University School of Public Health, Providence, RI, USA (K B Biello PhD); Department of Infectious Diseases, Harvard Medical School and Beth Israel Deaconess Medical Center, Boston, MA, USA (Prof K H Mayer); Department of Global Health and Population, Harvard T H Chan School of Public Health, Boston, MA, USA (Prof K H Mayer); Humsafar Trust, Mumbai, India (S Rawat, A Dange, V Anand, D Baruah); Department of Psychiatry, Harvard Medical School, Massachusetts General Hospital, Boston, MA, USA (C A Bedoya PhD, C O’Cleirigh); Sahodaran, Chennai, India (S Menon); Department of Psychiatry and Human Behaviour, Alpert Medical School, Brown University, Providence, RI, USA (L Klasko-Foster PhD); Department of Epidemiology, UCLA Fielding School of Public Health, Los Angeles, CA, USA (Prof M J Mimiaga); Department of Psychiatry and Biobehavioural Sciences, UCLA David Geffen School of Medicine, Los Angeles, CA, USA (Prof M J Mimiaga)

## Abstract

**Background:**

Men who have sex with men (MSM) in India are extremely marginalised and stigmatised, and therefore experience immense psychosocial stress. As current HIV prevention interventions in India do not address mental health or resilience to these stressors, we aimed to evaluate a resilience-based psychosocial intervention in the context of HIV and sexually transmitted infection (STI) prevention.

**Methods:**

We did a multicity, randomised, clinical efficacy trial in Chennai (governmental tuberculosis research institute) and Mumbai (non-governmental organisation for MSM), India. Inclusion criteria were MSM, aged 18 years or older, who were at risk of HIV acquisition or transmission, defined as having any of the following in the 4 months before screening: anal sex with four or more male partners (protected or unprotected), diagnosis of an STI, history of transactional sex activity, or condomless anal sex with a man who was of unknown HIV status or serodiscordant. Participants were required to speak English, Tamil (in Chennai), or Hindi (in Mumbai) fluently. Eligible individuals were randomly assigned (1:1) to either a resilience-based psychosocial HIV prevention intervention, consisting of group (four sessions) and individual (six sessions) counselling alongside HIV and STI voluntary counselling and testing, or a standard-of-care control comprising voluntary counselling and testing alone. The primary outcomes were number of condomless anal sex acts with male partners during the past month (at baseline and 4 months, 8 months, and 12 months after randomisation), and incident bacterial STIs (at 12 months after randomisation). Resilience-related mediators included self-esteem, self-acceptance, and depression. Recruitment is now closed. This trial is registered with ClinicalTrials.gov, NCT02556294.

**Findings:**

Between Sept 4, 2015, and June 28, 2018, we enrolled 608 participants; 305 (50%) were assigned to the psychosocial intervention condition and 303 (50%) were assigned to the control condition. 510 (84%) of 608 men completed an assessment at 4 months after randomisation, 483 (79%) at 8 months, and 515 (85%) at 12 months. 512 (99%) of 515 men had STI data from the 12-month assessment. The intervention condition had a 56% larger reduction in condomless anal sex acts (95% CI 35–71; p<0.0001) from baseline to 4-month follow-up, 72% larger reduction (56–82; p<0.0001) from baseline to 8-month follow-up, and 72% larger reduction (53–83; p<0.0001) from baseline to 12-month follow-up, compared with the standard-of-care control condition (condition by time interaction; χ^2^=40.29, 3 df; p<0.0001). Improvements in self-esteem and depressive symptoms both mediated 9% of the intervention effect on condomless anal sex acts. Bacterial STI incidence did not differ between study conditions at 12-month follow-up.

**Interpretation:**

A resilience-based psychosocial intervention for MSM at risk of HIV acquisition or transmission in India was efficacious in reducing condomless anal sex acts, with evidence for mediation effects in two key target resilience variables. HIV prevention programmes for MSM in India should address mental health resilience to augment reductions in the risk of sexually transmitted HIV.

**Funding:**

National Institute of Mental Health.

## Introduction

India, with one third of the world’s population of HIV-positive individuals,^1^ reported greater than 87 000 new HIV infections in 2017 (the most recent available data).^2^ The Indian National AIDS Control Organization considers men who have sex with men (MSM) to be a core risk group for HIV, with an estimated prevalence of 4.3%; more than 16 times higher than the national average of 0.3%.^2^ However, some studies have shown that the prevalence might be even higher among some MSM. For example, a study including 12 cities in India found a weighted HIV prevalence of 7.0% among MSM, with an estimated annual incidence of 2.2%.^3^ Given the large population size of India (more than 1.2 billion people),^4^ it could have one of the largest concentrations of MSM of any country in the world. As such, MSM in India are an important population to intervene in, to decrease the global burden of HIV and AIDS.

MSM in India are still, largely, a hidden population, facing unique and complicated psychosocial challenges which have a negative effect on mental health. Some of these challenges include stigmatisation; discrimination; internalised homophobia; trouble in coming out (disclosing one’s sexuality to others); familial and societal pressure to get married to a woman and have children; leading a double life; pressure to keep sexual minority status a secret; difficulties with sexual relationships; fears of getting infected with HIV; monetary difficulties; violence; harassment from police, thugs, the community, family, and in health-care settings; and elevated rates of childhood sexual abuse, alcohol use, depression, and suicidality,^5–11^ with varying associations between these variables and HIV acquisition or transmission risk behaviours. Taken together, similar to the syndemic context of HIV risk described previously,^12–15^ HIV acquisition and transmission risk behaviours in India occur within the context of intertwined syndemic problems, meaning that the risk behaviours or poor health behaviours exhibited by MSM occur within the context of all of these issues interacting with each other.^16^ One study that specifically examined syndemics in Indian MSM found additive effects of five syndemic conditions (depression, illicit drug use, alcohol dependence, interpersonal violence, and childhood sexual abuse) on unprotected anal intercourse, and synergistic effects of interpersonal violence and depression.^17^ Another study found evidence for additive and interaction effects of violence or victimisation, drug use, and frequent alcohol use on inconsistent condom use.^18^

Despite the number of studies which have found that HIV risk is increased in the context of syndemic psychosocial problems among MSM, very few studies worldwide, and even fewer in India, address either individual psychosocial problems or syndemics with behavioural HIV prevention interventions. With respect to individual syndemic problems, in the USA, O’Cleirigh and colleagues^19^ found that treating cognitions related to childhood sexual abuse, together with HIV risk reduction counselling and testing, was superior to HIV risk reduction counselling and testing alone. Mimiaga and colleagues found that addressing problematic stimulant use with behavioural activation was feasible and acceptable,^20^ and superior to HIV risk reduction counselling and testing alone.^21^ With respect to a number of syndemic problems related to sexual minority stress, Pachankis and colleagues^22^ trialled a transdiagnostic treatment, and found that it was superior to a waiting-list control. One study in India, with a pre-test and post-test design, found initial evidence for an effect of a syndemic theory-based intervention on condomless anal sex among MSM.^23^ Generally, however, most government-funded interventions in India for MSM only involve condom distribution and peer sexual health education,^24^ and there have been some studies of similar or creative approaches to this educational intervention,^25^ and HIV voluntary counselling and testing.^26^

To address this gap in interventions, we developed a culturally tailored,^9^ transdiagnostic, unified intervention that addresses both psychosocial risk and HIV acquisition and transmission risk for Indian MSM. The intervention is based on a conceptual model that involves fostering resilience to psychosocial and HIV risk by addressing various components of sexual minority stress that are specific to India, and building self-esteem and self-acceptance. It contains both a group component and individual counselling. In a preliminary trial with 96 participants in Chennai, India, we found evidence for participant acceptability and trial feasibility, as well as a significant difference in the rate of decrease in condomless anal sex acts favouring the intervention compared with voluntary HIV and sexually transmitted infection (STI) counselling and testing alone.^27^ However, generalisability was poor because it was done in Chennai only, with a modest sample size, leading to low power to detect differences in primary outcomes and hypothesised mediators. Therefore, in this study we aimed to do a more generalisable, multicity, fully powered, randomised, controlled, efficacy trial of this psychosocial intervention, in the context of sexual health.

## Methods

### Study design and participants

We did a two-arm, multicity, randomised, controlled, efficacy trial at the National Institute for Research in Tuberculosis (NIRT, formally the Tuberculosis Research Centre) in Chennai, India and the Humsafar Trust (an LGBTQ+ non-governmental organisation that provides services to sexual minority individuals) in Mumbai, India. Inclusion criteria were MSM, aged 18 years or older, who were at risk of HIV acquisition or transmission, defined as having any of the following in the 4 months before screening: anal sex with four or more male partners (protected or unprotected), diagnosis of an STI, history of transactional sex activity, or condomless anal sex with a man who was of unknown HIV status or serodiscordant. Participants were either kothi (feminine acting, usually the receptive sexual partner) or double-decker (either insertive or receptive sexual partner; usually has male and female partners) at screening, and were required to speak English, Tamil (in Chennai), or Hindi (in Mumbai) fluently. Exclusion criteria were age younger than 18 years; unable or unwilling to provide informed consent; any evidence of active, unstable, major mental illness (ie, untreated psychosis or mania) or any primary psychotic disorder, even if treated; and being deemed by the local principal investigator or study outreach staff to be engaging in deception about inclusion criteria. Eligible participants provided written informed consent before being enrolled. Institutional review board approval was granted at NIRT, Humsafar Trust, and Partners Health Care (Massachusetts General Hospital, Boston, MA, USA). The trial was also approved by the India Council of Medical Research Health Ministry Screening Committee, and by the India National AIDS Control Organization. Further details on the methods are described in our protocol paper.^28^

### Randomisation and masking

Eligible individuals were randomly assigned (1:1) to either a resilience-based psychosocial HIV prevention intervention, consisting of group (four sessions) and individual (six sessions) counselling alongside HIV and STI voluntary counselling and testing, or a standard-of-care control comprising voluntary counselling and testing alone. Randomisation was done in rotating blocks of two, four, and six, via Randomize.net,^29^ and the experimental intervention began when a group of eight participants were assigned to that study arm. Study staff were completely masked to the randomisation until the completion of the visit.

### Procedures

All participants, regardless of study condition, received standard-of-care pre-test and post-test counselling with testing for HIV and STIs at their baseline and 12-month follow-up visits. The approach was consistent with but augmented the Indian National AIDS Control Organization guidelines.^30^

In addition to HIV and STI counselling, the experimental psychosocial intervention addressed resilience by increasing self-acceptance and self-esteem while decreasing distress in the form of depression,^9,31^ and was previously tested in a pilot randomised trial in Chennai.^27^ The intervention included a group counselling component with groups of eight participants (four sessions) to reduce feelings of isolation, facilitate peer guidance on challenging situations faced on account of one’s sexual orientation, and provide social support for similar potentially stigmatising issues. Facilitators for the groups were one master’s-level counsellor and one peer interventionist. Each session was manualised and focused on various topics related to fostering resilience: introductions and self-acceptance; safer ways to meet men, and guidance on alcohol or substance use and misuse; HIV and STI (health risks) vulnerability and education; and risk-reduction skills and looking towards the future.

As well as the group sessions, the intervention included six individual counselling sessions delivered by a master’s-level counsellor, which addressed case management needs and allowed for counselling that was more directly related to personalised risk-reduction goals that might best be addressed in a private, one-to-one format. Specific topics of the individual sessions included: self-esteem and coming out experience (session one), sexual risk limits (session two), risk education and reduction, and problem-solving (sessions three, four, and five), and overview of their individual goals (session six). The timeframe was to complete these sessions before the 4-month assessment.

Before participant enrolment began, counsellors and peer interventionists at each site participated in an initial 3-day training with the principal investigators, followed by additional training given locally by the site principal investigators. As the Chennai site had previously tested the intervention as part of the pilot study, the Mumbai site piloted the intervention (data not included in this study) with one group of participants (n=8) and received weekly supervision during this pilot phase. The two site teams then participated in a supervision call with the US team once every 2 weeks throughout the trial, which included feedback from transcripts of the sessions, and weekly meetings led by the site principal investigator. Fidelity of the intervention was assessed by reviewing a random 10% of the audio-recorded counselling sessions; there was high adherence (95% individual and 94% group) and competence (93% individual and 96% group) across the two study sites.^32^

There were four major assessments completed via both interviewer-assisted and self-assisted audio-computer interviews on a study tablet computer: baseline assessment (before randomisation) and 4-month (considered to be post-intervention), 8-month, and 12-month follow-up assessments. Participants were compensated with between 250 and 1000 Indian Rupees (approximately US$3.30–$13.20) for each study visit, to cover expenses related to transportation reimbursement and food. At baseline, participants self-reported their age, MSM identity, religion, level of education, and employment status. We also assessed history of HIV and STI testing, symptoms, and diagnoses.

At each assessment, participants completed a seven-item questionnaire via audio-computerised self-assessment that we developed on the basis of our pilot study.^27^ The items included number of male partners, number of times they had anal sex with male partners, and condom use with male partners. Because pre-exposure prophylaxis (PrEP) is not generally available for the population included in this study, it was not accounted for in the present analyses, and we believe that none of the participants in the trial were taking PrEP during the study.

Participants were tested for HIV and bacterial STIs at baseline and at their 12-month follow-up visits. Details of testing were described previously.^28^ In brief, participants received a rapid HIV test, and all reactive samples were confirmed with further testing. For syphilis, serum samples were tested initially by using rapid plasma reagin, and reactive sera were tested for antibodies to confirm *Treponema pallidum* (syphilis) infection. To test for urethral chlamydia and gonorrhoea, participants provided a small urine sample which was assessed via nucleic acid amplification testing (NAAT); a laboratory staff member or trained clinician took an oropharyngeal sample and a rectal swab, also assessed via NAAT. At the NIRT, all participants with diagnosed STIs received treatment, and anyone who tested positive for HIV was referred to the government HIV and AIDS programme clinics where treatment was provided free of charge. At the Humsafar Trust, presumptive treatment for gonorrhoea and chlamydia was administered as per national guidelines, and for syphilis, participants were referred to a government hospital for free treatment.

Incident HIV infection was defined as a non-reactive test at baseline and a reactive test at 12-month follow-up. For chlamydia and gonorrhoea, all reactive tests at 12-month follow-up were presumed to be incident as all participants with a reactive test at baseline received treatment to cure. For syphilis, an incident infection was defined as a non-reactive test at baseline and reactive test at 12-month follow-up, or reactive at baseline but with a four-fold increase in the titre between the baseline and 12-month visits. Test results were adjudicated by co-author KHM.

### Outcomes

The primary outcomes were number of condomless anal sex acts with male partners during the past month (at baseline and 4 months, 8 months, and 12 months after randomisation) and incident bacterial STIs (assessed only at the 12-month follow-up visit).

On the basis of our theoretical framework, we assessed a priori hypothesised mediators of the intervention effect, including depression, measured using the validated Center for Epidemiologic Studies Depression Scale (CES-D-10);^33^ self-esteem, measured using the validated Rosenberg Self-Esteem Scale;^34^ and self-acceptance, measured by asking participants to report on a scale from 1 to 10, “how much do you accept yourself for who you are no matter what other people think?”. Given the hybrid efficacy and effectiveness design, we examined whether implementation site (Mumbai *vs* Chennai) modified the intervention effect as a prespecified analysis done after the primary analysis.

### Statistical analysis

The power calculation for the primary outcomes was based on our pilot trial, and assumed 21% incidence of STIs in the control arm and 16% in the intervention condition, resulting in the ability to detect a 30% (or greater) difference in the 12-month STI incidence. Similarly, we assumed a 25% greater rate of change in condomless anal sex acts across the 4-month, 8-month, and 12-month assessments (baseline to 12-month follow-up) in the intervention condition than in the control condition. Based on these assumptions, with an α level of 0.05 and a power of 90% for each, we estimated that we needed 480 participants to complete the study. To account for attrition, for which we assumed a rate of 20% between the baseline assessment and 12-month follow-up, we estimated that 600 individuals needed to be enrolled. However, because the intervention groups each had to include eight participants, we sought to randomise 608 participants to have a multiple of 16.

Means with SDs and proportions for baseline characteristics are reported overall and by study condition. Differences in characteristics by study condition were examined using *t* tests for continuous measures and χ^2^ tests for nominal measures. We did complete-case analyses, so no data were imputed.

For condomless anal sex acts, we examined the distribution of responses before data analysis. To correct for extreme outliers, we winsorised the data to the 1st and 99th percentile for each visit.^35^ Because number of condomless anal sex acts is count data and these were not normally distributed, we estimated mean counts to examine differences by study condition using a mixed-effects model (to account for within-person correlation), specifying a negative binomial distribution and log link with a random intercept and slope for month of follow-up (baseline and 4, 8, or 12 months). Notably, the primary interpretation of the findings was robust to model selection. Given that our aim was to examine differences in the changes in number of condomless anal sex acts from baseline to follow-up, the models contained terms for study condition, time, and their interaction; differences between study arms in the change of number of condomless anal sex acts from baseline to follow-up indicates a significant effect of the interaction. Model coefficients were exponentiated for interpretation as incidence rate ratios (IRRs).

Incident infections were calculated as the proportion of the sample at 12-month follow-up who had a new infection. χ^2^ tests were then used to examine differences in the proportion of incident HIV and STIs between study arms.

To assess for effect of site, an interaction term was added to all main effects models. For significant interactions, IRRs stratified by site are reported.

To assess for mediation variables, we did a multiple structural equation model (MSEM) analysis in Mplus version 8.3 using maximum likelihood estimation. MSEM accommodates the test of multiple mediation pathways with repeated measures by allowing for the calculation of separate within-level (within a participant over time) and between-level (between participants) components of indirect effects.^36^ The tested model examined the simultaneous influence of the study condition on participants’ depression, self-esteem, and self-acceptance at 4, 8, and 12 months after randomisation (path A) and the influence of changes to participants’ reported depression, self-esteem, and self-acceptance on the number of condomless anal sex acts reported at 4, 8, and 12 months (path B). Self-esteem and self-acceptance scales were reverse coded to avoid inconsistent mediation effects (ie, higher scores signify lower self-esteem and self-acceptance). We used Satorra-Bentler χ^2^ difference testing to compare the restrictive model without potential mediators to the final mediated model.^37^ Moreover, the indirect effects (A × B) were estimated using the model constraint command to decompose the total effect into a direct effect of the exposure on the outcome (ie, the effect of study condition on number of condomless anal sex acts) and individual indirect effects operating through the hypothesised pathways (depression, self-esteem, and self-acceptance). Indirect effects with CIs that do not include 0 imply a significant mediated effect. Intraclass correlation coefficients were calculated with the addition of each potential mediator to estimate the proportion of the variance in condomless sex explained by between-person differences. This trial is registered with ClinicalTrials.gov, NCT02556294; recruitment is now closed.

### Role of the funding source

The funder of the study had no role in study design, data collection, data analysis, data interpretation, or writing of the report.

## Results

**Figure 1:**
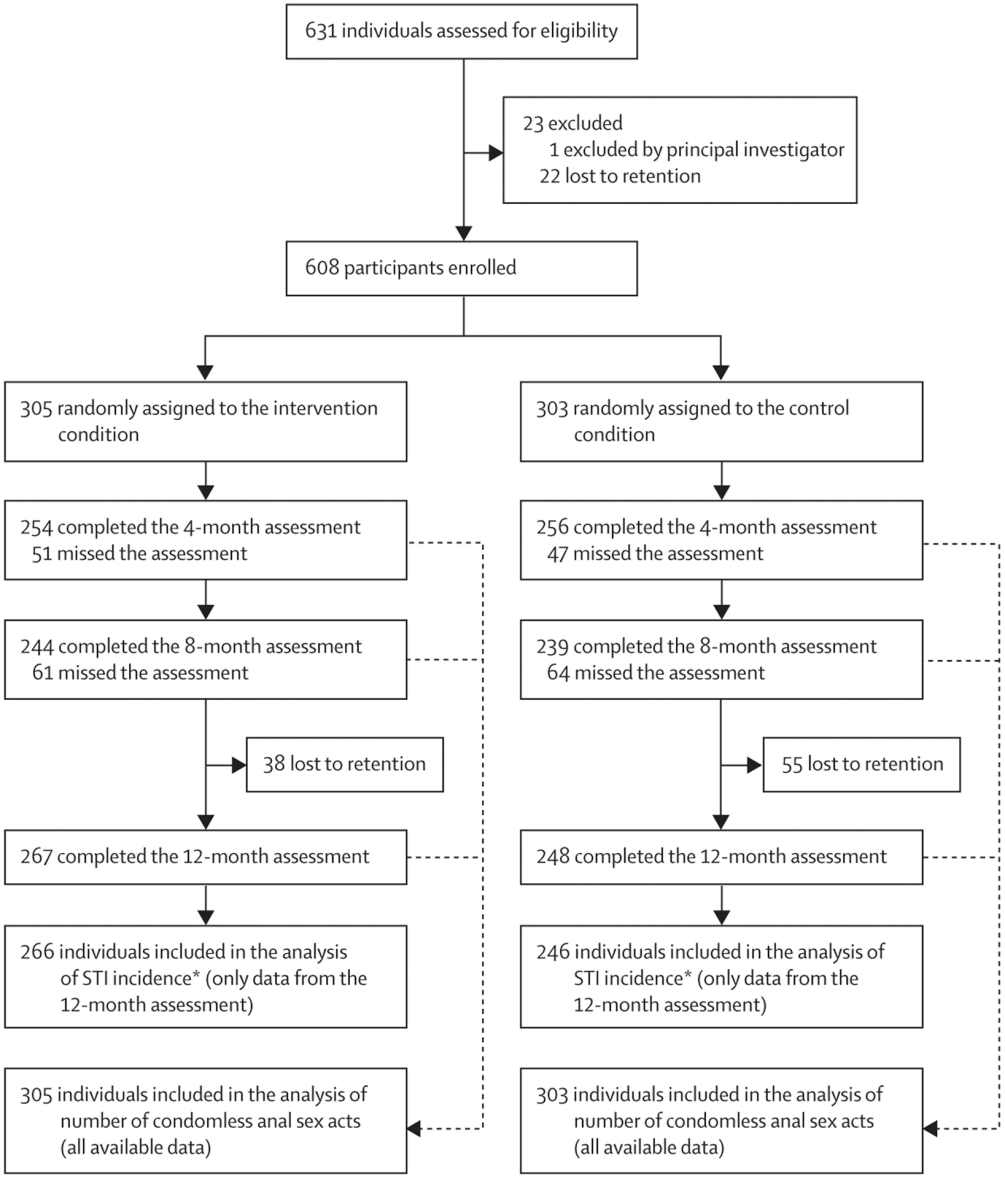
Trial profile *512 (99%) of 515 men who completed the 12-month assessment had STI incidence data available. STI=sexually transmitted infection.

Between Sept 4, 2015, and June 28, 2018, we enrolled 608 participants who were randomly assigned to the intervention condition (n=305) or the control condition (n=303; figure 1, table 1). The last follow-up visit was on Aug 30, 2019. Of the 608 participants, 510 (84%) completed an assessment at 4 months, 483 (79%) at 8 months, and 515 (85%) at 12 months. 512 (99%) of 515 men had STI data from their 12-month assessment. Attrition did not differ by study condition, and aligned with our assumptions in our sample size calculations. We were aware of only one couple that entered the trial; however, they were, by chance, both assigned to the control condition.

Participants in the intervention condition attended a mean of 4.8 individual sessions (SD 1.8); 204 men (67%) attended all individual sessions and 256 (84%) attended at least half (three or more sessions). Participants attended a mean of 2.5 group sessions (SD 1.5); 113 men (37%) attended all group sessions and 223 (73%) attended at least half (two or more sessions).

At baseline, the mean number of condomless anal sex acts in the past month was 8.1 (SD 18.3).

Details about baseline STIs are described elsewhere,^38^ but, in brief, at baseline, 58 (10%) of 608 men were HIV-positive (21 men were known positive, 37 tested positive and were previously unknown status). Additionally, at baseline, 199 men (33%) tested positive for at least one bacterial STI.

**Figure 2:**
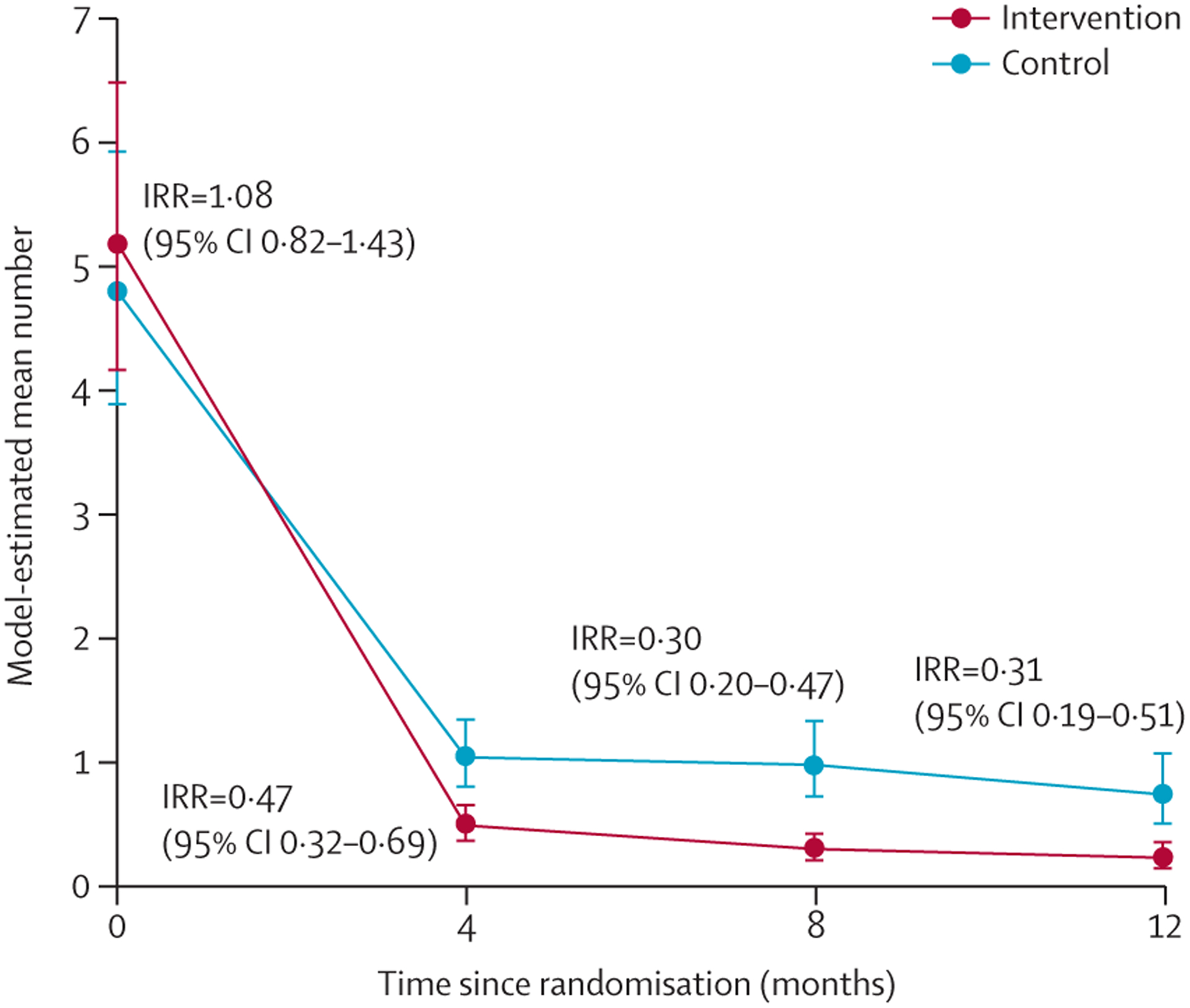
Change in number of condomless anal sex acts over 12 months, by study condition IRR=incidence rate ratio.

Based on model-estimated means, the baseline number of condomless anal sex acts did not differ between the intervention and control conditions (mean 5.2 [SE 0.5] in the intervention condition *vs* 4.8 [0.5] in the control condition; p=0.58). There was a significant effect of time, with reductions in number of condomless anal sex acts from baseline to 4, 8, and 12 months of follow-up across both study arms (F=44.65, 2/68 df, p<0.0001). The intervention condition had a 56% larger reduction (95% CI 35–71; p<0.0001) in condomless anal sex acts from baseline to the 4-month follow-up, 72% larger reduction (56–82; p<0.0001) from baseline to 8-month follow-up, and 72% larger reduction (53–83; p<0.0001) from baseline to 12-month follow-up, compared with the control condition (χ^2^=40.29, 3 df, p<0.0001). Moreover, at 4, 8, and 12 months of follow-up, the mean number of condomless anal sex acts was significantly lower in the intervention condition than in the control condition (all p values <0.0001; figure 2).

At the 12-month assessment there was, overall, a 22% incidence of any bacterial STI, and incidence did not differ between study conditions (p=0.50). The incidence of specific STIs and of HIV infection also did not differ between study conditions (table 2).

**Figure 3:**
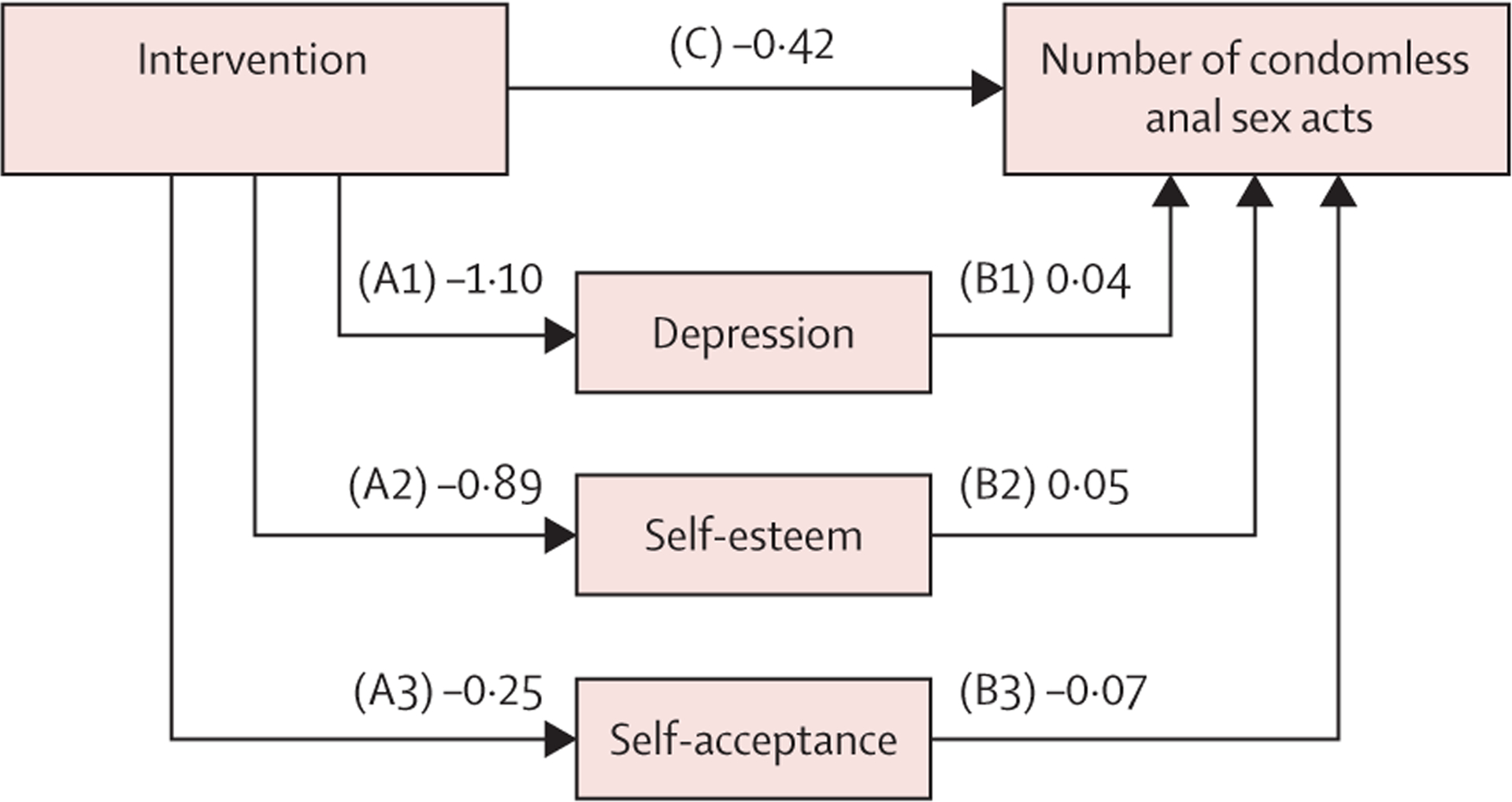
Final mediation model with parameter estimates The intervention effect pathways are depicted as follows: effect of intervention on depression (path A1), effect of intervention on self-esteem (A2), effect of intervention on self-acceptance (A3), effect of depression on number of condomless anal sex acts (B1), effect of self-esteem on number of condomless anal sex acts (B2), effect of self-acceptance on number of condomless anal sex acts (B3), and direct effect of intervention on number of condomless anal sex acts (C).

Intraclass correlation coefficients ranged from 0.22 to 0.39 (all p values <0.0010), suggesting that at least one quarter of the variability in condomless anal sex acts was attributable to between-person differences for each mediator, supporting the mediation analysis. Moreover, the mediated model showed improved fit (χ^2^ (22 df)=27693.18, p<0.0010) compared with the model without mediators. The intervention significantly reduced depression (β=−1.10, p=0.0030) and improved self-esteem (β=−0.89, p=0.0010) and self-acceptance (β=−0.25, p=0.018) over time, and depression, self-esteem, and self-acceptance were all associated with number of condomless anal sex acts (path B; table 3, figure 3). Depression (β=−0.04, p=0.012) and self-esteem (β=−0.06, p=0.0060) were significant mediators; depression accounted for 8.5% of the mediated effect (95% CI 1.1–15.7) and self-esteem accounted for 9.2% (2.0–16.5). The indirect effect of the intervention on condomless anal sex acts through self-acceptance was not significant (β=0.02, p=0.10). Notably, an intervention effect on condomless anal sex acts remained after accounting for the mediation (β=−0.49, p<0.0010; path C).

The intervention effect on condomless anal sex acts or bacterial STIs did not differ by study site, and hence these effects were not stratified by site. However, there was a significant interaction between study site and intervention condition for HIV incidence (p=0.016). In Chennai, HIV incidence was significantly lower in the intervention condition than in the control condition (0% *vs* 4%; p=0.033); whereas in Mumbai there was no significant difference between arms (8% *vs* 4%; p=0.26).

## Discussion

This study showed that a resilience-based psychosocial intervention for MSM in India was successful in terms of reducing condomless anal sex acts with male partners, and that two of the hypothesised proximal intervention targets, self-esteem and depressive symptoms, mediated this effect in the expected direction. Though number of condomless anal sex acts differentially changed between study arms, STI incidence did not differ, and one of the targets of the intervention, the single-item assessment of self-acceptance, was not significant as a mediator. From the perspective of mental health and quality of life, these are important findings, especially given the evidence of disproportionately high levels of mental health distress seen in this disenfranchised and often hidden and overlooked population.^8,16,39^ To our knowledge, this study is the first to show that addressing resilience to the many barriers that Indian MSM face can reduce distress and an important health-risk behaviour, condomless anal sex with male partners.

However, from an HIV and STI prevention perspective, the absence of a significant difference in incidence between conditions, and the generally high level of incident STIs (23%) and HIV (4%) in this population are concerning. Although the risk behaviours for HIV, which were reduced in terms of self-reporting of condomless anal sex reductions in both study conditions, and STIs are not identical, incident STIs are a good biomarker of condomless oral and anal sex, and hence it is difficult to discern the degree to which self-report could be influenced by other variables. Similarly, syphilis can be transmitted through genital-to-genital rubbing, and does not necessarily require unprotected anal sex for transmission. The high HIV and STI rates at 12 months of follow-up show the great need for additional intervention components to reduce STIs in MSM in India. Furthermore, if and when PrEP becomes available on a large scale in MSM in India, the high STI rates suggest that use of PrEP will not obviate the relevance of counselling and skills-building around condom use, given the high prevalence of STIs.

The results of the mediational analyses provide validity for the conceptual model and hypothesised targets underlying the resilience-based intervention, related to the condomless anal sex outcome. The intervention model was based on building psychosocial resilience by increasing self-worth and decreasing distress,^9^ and therefore allowing participants to be more open to benefit from HIV risk-reduction counselling and messaging. We found that, in the multiple mediational model, self-esteem and depressive symptoms significantly mediated the intervention effect. The single-item indicator for self-acceptance did not emerge as significant in the mediational model, although the specific pathways (intervention to increases in self-acceptance, self-acceptance to decreases in condomless anal sex acts) were significant. The reasons for this absence of significance are not clear; although the indicator for self-acceptance was developed based on our pilot work and has face validity, it does not have the same level of empirical support with respect to measurement validity as does the Rosenberg self-esteem variable, which did show mediation and assesses a similar construct, and it was a single item.

Study site did not affect the primary outcomes (number of condomless anal sex acts or STI incidence). However, for the moderation analysis for HIV incidence, there was an effect, showing reduced HIV incidence in the intervention condition in Chennai, but not in Mumbai. This result should be interpreted with caution, as it was secondary, and the overall effects of the intervention were not powered to show an HIV incidence outcome. However, given the significance despite a-priori power, it could be an area for hypothesis generation.

This was a full-scale randomised controlled trial, and therefore had many elements in the methods related to rigour and potential replicability. These elements included the block randomisation, the use of interviewer-assisted and self-assisted audio-computer interviews and the assessment of condomless anal sex acts, the procedures for counsellor and peer-interventionist feedback and supervision, the examination of intervention targets or mediators, and the use of a biological primary outcome (STI incidence). However, this study also had several limitations. First, although we used interviewer-assisted and self-assisted audio-computer interviews to mitigate bias in self-reporting of condomless anal sex acts, there was potential for results related to demand characteristics (eg, people in the intervention condition artificially reducing the degree of self-reported condomless anal sex acts). That said, the measure assesses specific behaviours widely used in prevention trials, and the use of interviewer-assisted and self-assisted audio-computer interviews in a private setting might have mitigated the concern for differential reporting. Further, we hope that the potential bias of self-reporting was mitigated by providing HIV and STI counselling and testing at the baseline and 12-month assessment, as well as additional counselling and treatment when STIs were diagnosed at either visit. Participants in both arms reported reductions in condomless anal sex acts, but the intervention condition had greater reductions. This finding of a difference also occurring in the control condition is common in behavioural interventions where engagement in a research study can act as a minimal intervention itself. The assessment of self-acceptance was potentially not valid in that we did not have a validated scale, and hence used a single-item indicator. Additionally, the Mumbai site is a well known non-governmental organisation that provides extensive services to sexual minorities, and participation in the study required visits to their offices; those who chose to participate through such a site might not be representative of the broader population of Indian MSM. Generalisability would have been further enhanced if there were more than two sites, as additional geographical differences might exist across the multiple geographical zones in India. Group participation was less than ideal, which was somewhat expected given the marginalised population and difficulties in finding a time that everyone could meet. Notably, a post-hoc compliance-adjusted causal analysis with inverse probability weighting suggested that the effect sizes were stronger when accounting for compliance, but the interpretation did not change meaningfully. Future efforts with alternative ways of holding the group sessions (ie, via mobile phone) or ways to bolster attendance are needed. Finally, attrition ranged from 15% to 21% for the follow-up visits, which could have biased the results if those who were retained differed from those lost to follow-up in important ways. Importantly, this level of attrition was expected (and we powered our analyses on this assumption) and is common in interventional studies with 12-month follow-up. Because the rates of attrition did not differ by study condition, it is most likely that the observed results would be biased toward a null effect, suggesting that our effect sizes could be underestimated. As such, attrition might have led to more conservative estimates of the true effect.

This study found that integrating counselling to reduce condomless anal sex, with counselling focused on resilience to psychosocial stress, is a valid and culturally acceptable way to provide intervention to MSM in India who face tremendous psychosocial stressors. Generally, hypothesised intervention targets using validated measures (eg, self-esteem, depression) were shown to mediate the outcome, condomless anal sex acts. However, there was not an effect on biological outcome, bacterial STI or HIV incidence. These results, together with our previous work^9,27^ indicate that as various prevention programmes are implemented in India, it is important and feasible to include content related to self-worth and resilience in these interventions. A concurrent analysis of the cost-effectiveness of this intervention is pending and will help determine the usefulness of disseminating and implementing it in other settings across India. The India National Programme for HIV prevention among MSM might include interventionist counsellors that do not have master’s degrees, and hence implementation trials or task-shifting approaches are needed. This study, together with additional previous studies, documenting mental health concerns among MSM in India, highlight the need to integrate mental health and psychosocial aspects in targeted interventions for key populations, such as MSM, in India. Additionally, as PrEP is scaled up in India for this population, it will be important to study or utilise components of this approach with its implementation, and additional content related to STI prevention, given the high rates of STI incidence found in this trial.

## Figures and Tables

**Table 1:** Baseline characteristics

	Total (n=608)	Intervention (n=305)	Control (n=303)
Age, years	26.2 (6.3)	26.5 (6.5)	26.1 (6.0)
Number of male sexual partners*	11.9 (46.3)	11.5 (29.7)	9.2 (13.9)
Number of condomless anal sex acts with male partners*	8.1 (18.3)	8.7 (20.8)	7.5 (15.3)
Depression score (CES-D-10; range 0–30)	10.7 (6.5)	10.6 (6.7)	10.9 (6.2)
Self-acceptance score (range 1–10)	8.4 (2.0)	8.4 (2.1)	8.5 (1.9)
Self-esteem score (Rosenberg Scale;range 0–30)	21.4 (4.8)	21.3 (4.8)	21.5 (4.8)
Subpopulation identity†			
Kothi	270 (44%)	139 (46%)	131 (43%)
Double-decker	202 (33%)	96 (31%)	106 (35%)
Gay or other	135 (22%)	69 (23%)	66 (22%)
Religion			
Hindu	441 (73%)	219 (72%)	222 (73%)
Muslim	79 (13%)	41 (13%)	38 (13%)
Christian	57 (9%)	32 (10%)	25 (8%)
Other, agnostic, or atheist	31 (5%)	13 (4%)	18 (6%)
Level of education			
Graduate or professional degree	86 (14%)	47 (15%)	39 (13%)
College degree	146 (24%)	80 (26%)	66 (22%)
Higher secondary	147 (24%)	71 (23%)	76 (25%)
Secondary	122 (20%)	58 (19%)	64 (21%)
Less than secondary	107 (18%)	49 (16%)	58 (19%)
Employment status			
Full time	294 (48%)	148 (49%)	146 (48%)
Part time	98 (16%)	51 (17%)	47 (16%)
Unemployed or other	216 (36%)	106 (35%)	110 (36%)
Marital status			
Married to a woman	38 (6%)	27 (9%)	11 (4%)
Not married to a woman	546 (90%)	266 (87%)	280 (96%)
Children			
Yes	47 (8%)	29 (10%)	18 (6%)
No	561 (92%)	276 (90%)	285 (94%)
STI test in the past 4 months			
Yes	56 (9%)	32 (10%)	24 (8%)
No	547 (91%)	271 (89%)	276 (92%)
STI symptoms in the past 4 months			
Yes	142 (23%)	73 (24%)	69 (23%)
No	466 (77%)	232 (76%)	234 (77%)
Any previous HIV test			
Yes	389 (64%)	195 (64%)	194 (64%)
No	219 (36%)	110 (36%)	109 (36%)
Self-reported STI diagnosis in the past 4 months			
Yes	20 (3%)	11 (4%)	9 (3%)
No	586 (97%)	293 (96%)	293 (97%)
HIV-positive test at baseline			
Yes	58 (10%)	32 (10%)	26 (9%)
No	550 (90%)	273 (90%)	277 (91%)
Positive for any bacterial STI at baseline			
Yes	199 (33%)	92 (30%)	107 (35%)
No	409 (67%)	213 (70%)	196 (65%)

Data are mean (SD) or n (%). Participants who answered “do not know” were treated as missing for that question. CES-D-10=Center for Epidemiologic Studies Depression Scale. STI=sexually transmitted infection.

* Self-reported sexual behaviour during the past month.

† Identity in this population is fluid, so some participants identified one way at screening (eg, kothi) and another way on the baseline assessment after enrolment at randomisaton (eg, gay); hence data are based on identity at screening.

**Table 2:** STI and HIV incidence at 12-month follow-up, overall and by study condition

	Overall	Intervention	Control	p value
Chlamydia (n=489)	67 (14%)	38 (15%)	29 (12%)	0.42
Gonorrhoea (n=494)	43 (9%)	21 (8%)	22 (9%)	0.66
Syphilis (n=512)	29 (6%)	14 (5%)	15 (6%)	0.68
Any bacterial STI* (n=512)	117 (23%)	64 (24%)	53 (22%)	0.50
HIV† (n=470)	18 (4%)	9 (4%)	9 (4%)	0.91

Data are n (%) or p values. STI=sexually transmitted infection.

* Any bacterial STI includes chlamydia, gonorrhea, and syphilis.

† HIV incidence was only measured among those who were negative at baseline.

**Table 3:** Effect estimates from a multilevel structural equation model analysis assessing intervention effects, including potential intervention mediators, on number of condomless sex acts, from baseline to 12-month follow-up

	Effect estimate (SE)	95% CI	p value	IRR (SE)	IRR 95% CI
**Between-person effects (over time)**					
Intervention to mediator (path A)					
Depression (path A1)	−1.10 (0.37)	−1.82 to −0.37	0.0030	0.33 (0.12)	0.10 to 0.58
Self-esteem (path A2)	−0.89 (0.27)	−1.43 to −0.36	0.0010	0.41 (0.11)	0.19 to 0.63
Self-acceptance (path A3)	−0.25 (0.11)	−0.46 to −0.04	0.018	0.78 (0.08)	0.61 to 0.94
Intervention to number of condomless anal sex acts (path C)	−0.42 (012)	−0.66 to −0.18	0.0010	0.66 (0.08)	0.50 to 0.82
**Within-person effects**					
Mediator to number of condomless anal sex acts (path B)					
Depression (path B1)	0.04 (0.01)	002 to 0.05	<0.0010	1.04 (0.01)	1.02 to 1.06
Self-esteem (path B2)	0.05 (0.01)	0.03 to 0.07	<0.0010	1.05(0.01)	1.03 to 1.07
Self-acceptance (path B3)	−0.07 (0.02)	−0.12 to −0.01	0.017	0.93 (0.03)	0.88 to 0.99
**Indirect effects**					
Intervention to mediator to number of condomless anal sex acts					
Depression	−0.04 (0.02)	−0.07 to−0.01	0.012	0.96 (0.02)	0.93 to 0.99
Self-esteem	−0.06 (0.02)	−0.08 to −0.01	0.0060	0.96 (0.02)	0.93 to 0.99
Self-acceptance	0.02 (0.01)	−0.01 to 0.04	0.10	1.01 (0.01)	0.99 to 1.04
**Total direct effect**					
Intervention to number of condomless anal sex acts (path C)	−0.49 (0.13)	−0.74 to.0.24	<0.0010	0.61 (0.08)	0.46 to 0.77

IRR=incidence rate ratio.

## Research in context

### Evidence before this study

In 2001, during a visit to India, the first author (SAS) met with a non-governmental organisation leader in Chennai, India (SM) who educated him about the situation for men who have sex with men (MSM) in India, a hidden population, and about the different identities seen in Chennai. We developed a proposal to survey MSM, and did literature searches for terms such as “MSM, India” in PubMed. Initially, in the early 2000s, there were few published papers, but this number increased with time. We published our own series of papers, and continued to stay abreast of the literature, periodically doing literature searches as studies emerged with expanding collaborators. This study is based on our own formative work and our pilot trial from 2005 onwards.

### Added value of this study

Despite the intense mental health and contextual variables related to being a man who has sex with men in India, HIV risk reduction programmes and published interventions do not address resilience to psychosocial stress. This study has added value because it addressed HIV risk behaviour among this population by simultaneously addressing important mental health and resilience variables in a population that has long been stigmatised and neglected.

### Implications of all the available evidence

As public health campaigns continue to emerge for MSM in India and other countries globally where sexual minorities are similarly stigmatised, interventions that simultaneously address resilience to psychosocial and societal stress in health behaviour are possible. The continued high incidence of sexually transmitted infections, however, further highlights the need for continued work with this population.
